# Transcriptomic Markers of Recombinant Human Erythropoietin Micro-Dosing in Thoroughbred Horses

**DOI:** 10.3390/genes12121874

**Published:** 2021-11-24

**Authors:** Anna R. Dahlgren, Heather K. Knych, Rick M. Arthur, Blythe P. Durbin-Johnson, Carrie J. Finno

**Affiliations:** 1 School of Veterinary Medicine, University of California Davis, Davis, CA 95616, USA; adahlgren@ucdavis.edu (A.R.D.); rmarthur@ucdavis.edu (R.M.A.); 2 K.L. Maddy Equine Analytical Pharmacology Lab and Department of Molecular Biosciences, School of Veterinary Medicine, University of California Davis, Davis, CA 95616, USA; hkknych@ucdavis.edu; 3 Genome Center, Bioinformatics Core Facility, University of California, Davis, CA 95616, USA; bpdurbin@ucdavis.edu

**Keywords:** RNA sequencing, biomarkers, rHuEPO, doping

## Abstract

Recombinant human erythropoietin (rHuEPO) is a well-known performance enhancing drug in human athletes, and there is anecdotal evidence of it being used in horse racing for the same purpose. rHuEPO, like endogenous EPO, increases arterial oxygen content and thus aerobic power. Micro-doping, or injecting smaller doses over a longer period of time, has become an important concern in both human and equine athletics since it is more difficult to detect. Horses offer an additional challenge of a contractile spleen, thus large changes in the red blood cell mass occur naturally. To address the challenge of detecting rHuEPO doping in horse racing, we determined the transcriptomic effects of rHuEPO micro-dosing over seven weeks in exercised Thoroughbreds. RNA-sequencing of peripheral blood mononuclear cells isolated at several time points throughout the study identified three transcripts (*C13H16orf54*, *PUM2* and *CHTOP*) that were significantly (*P_FDR_* < 0.05) different between the treatment groups across two or three time point comparisons. *PUM2* and *CHTOP* play a role in erythropoiesis while not much is known about *C13H16orf54*, but it is primarily expressed in whole blood. However, gene expression differences were not large enough to detect via RT-qPCR, thereby precluding their utility as biomarkers of micro-doping.

## 1. Introduction

Erythropoietin (EPO) is a protein predominantly secreted from the kidney to stimulate red blood cell (RBC) production in the bone marrow. As such, it increases hemoglobin mass, arterial oxygen content, and thus aerobic power [1]. Recombinant rHuEPO has been developed for clinical use to replace endogenous EPO in human patients suffering from various conditions associated with a decline in red blood cell counts [2]. It increases hemoglobin mass, arterial oxygen content, and thus aerobic power [2]. Recombinant human erythropoietin (rHuEPO) is also well known as a performance enhancing agent in human athletes [3,4,5]. Similar to training at higher elevations to naturally stimulate EPO [6], injecting rHuEPO has the same end result of enhancing an individual’s aerobic power [3]. This has led to its abuse in human sports and placement on the World Anti-Doping Agency’s list of prohibited substances [7].

In human sports, various methods of detecting rHuEPO administration have been employed. These include setting upper limits for hemoglobin and hematocrit levels [8], using isoelectric focusing (IEF) to detect differences in the charge profiling of endogenously versus exogenously produced EPO in urine and blood [9,10], and using sarcosyl polyacrylamide gel electrophoresis (SAR-PAGE) [11], to differentiate between exogenous and endogenous EPO by differences in migration, which are indicative of the small changes in molecular weight. For the latter two methods, the detection window used to be quite short, ranging from 24–85 h from the time of administration [9,11], but recent advances in detection methods have increased the detection window. An added challenge is the advent of micro-dosing, where small amounts of rHuEPO are administered over a long period of time to avoid detection [12]. However, with the improvements in IEF, SAR-PAGE, and sodium-dodecyl-sulfate polyacrylamide gel electrophoresis (SDS-PAGE) detection methodologies, micro-doses can be detected up to 72–104 h after administration [10,13]. Currently, the Athlete Biological Passport (ABP) regulates rHuEPO abuse and other blood doping agents in human athletes indirectly by monitoring for changes in hematological values such as hemoglobin, reticulocytes, and red blood cell count in an individual over time [12]. However, this screening method fails to detect micro-doses of rHuEPO [12]. Another alternative that has recently been investigated is using transcriptomic biomarkers to detect micro-dosing and supplement the ABP [14].

In Thoroughbred racehorses, there is anecdotal evidence of rHuEPO administration to enhance performance. A single small study has shown that rHuEPO administration does increase aerobic power and performance in horses, but these horses were splenectomized [15], which prevents horses from sustaining athletic performance [16]. There are no other studies to date that have evaluated the efficacy of rHuEPO administration, but methods of detection of rHuEPO administration have been evaluated in horses [17,18,19,20]. Routine testing of samples from equine athletes for detection of rHuEPO doping has been performed with liquid chromatography tandem mass spectrometry (LC-MS/MS) based assays [17,18], which are time consuming and cumbersome. Additionally, the detection window is short and micro-dosing is unlikely to be detected [17,18] since rHuEPO has a half-life of approximately 12.9 h in horses [19]. Monitoring hematological values over time, similar to the screening performed in human athletes, is not a viable option for detecting rHuEPO doping in horses due to their high RBC reserve and contractile spleen [16]. Many different methods of detection of rHuEPO administration have been evaluated in horses with similar times of detection. Enzyme-linked immunosorbent assays could detect doping 2–3 days after last administration [20], and a membrane assisted isoform immunoassay was able to detect rHuEPO 2–5 days after administration [21]. Digital droplet PCR and microfluidic quantitative PCR detected rHuEPO up to two days after administration [22,23]. An improved SAR-PAGE assay detected a single rHuEPO micro-dose for three days post administration [24]. High-Field Asymmetric waveform Ion Mobility Spectrometry (FAIMS), combined with LC-MS/MS and whole-genome resequencing, have also successfully detected rHuEPO doping, but the authors did not determine the time point at which the methods no longer detected rHuEPO [25,26]. However, none of the studies addressed micro-dosing detection and the bulk of the work optimizing testing methods were primarily *in vitro*. The studies that did administer rHuEPO to horses to test their methods did not use horses that were at a similar age or fitness level as racehorses and only one addressed micro-dosing detection.

To investigate the effects of rHuEPO micro-dosing in exercising Thoroughbred horses, we performed RNA-sequencing (RNA-seq) of peripheral blood mononuclear cells (PBMCs) in an experimental study. PBMCs were chosen as the cells of interest to identify transcriptomic changes since this cell population is already present in the blood samples that are routinely collected from racehorses. PBMCs are known to have EPO receptors that can be modulated by EPO treatment in humans [27]. Additionally, they are relatively easy to isolate and provide an abundance of RNA, making them useful to use in a commercial test. Thus, if a test for doping were developed, it would not require additional sampling. All horses were on an exercise schedule that simulated the workload of Thoroughbred racehorses. Our goal was to identify transcriptomic markers of rHuEPO micro-dosing to identify illicit doping of racehorses. We hypothesized that genes involved in erythropoiesis would be differentially expressed in micro-dosed horses, thereby providing a mechanism for testing micro-dosing in exercising Thoroughbreds.

## 2. Material and Methods

### 2.1. Horses

Five Thoroughbred mares, aged 4–7 years (median = 6), and five Thoroughbred geldings, aged 5–6 years (median = 5), were examined by a veterinarian and determined to be healthy. These ten horses were put on a consistent exercise protocol prior to and throughout the study. This protocol consisted of three days a week on an Equicizer (5 minutes’ (min) walk, 20 min trot, 5 min walk; Centaur Horse Walkers Inc., Mira Loma, CA, USA) and two days per week on a high speed treadmill (5 min walk at 1.6 m/s, then 5 min trot at 4 m/s, 5 min canter at 7 m/s and a cool out of 5 min walk at 1.6 m/s, then 5 min walk at 1.6 m/s, incline to 4% and 10 min trot at 4 m/s and then 5 min walk at 1.6 m/s; Mustang 2200, Graber AG, Switzerland). Once fit, the ten horses were again determined to be healthy by physical examination by a veterinarian, complete blood count, and serum biochemistry panel. The horses did not receive any other medication for at least two weeks prior to beginning the study.

**Figure 1:**
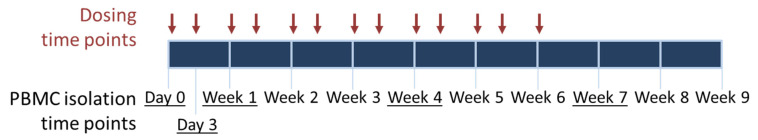
Schematic outlining time points of rHuEPO or saline dosing and blood draws for peripheral blood mononuclear cell (PBMC) isolations. The underlined time points indicate the time points that were submitted for RNA sequencing.

Six of the horses (3 mares, 3 geldings) were randomly assigned to the treatment group and the remaining four horses (2 mares, 2 geldings) to the saline control group. The horses in the treatment group received a subcutaneous injection of 20 IU/kg of rHuEPO (EPOGEN, Amgen, Thousand Oaks, CA, USA) with a sterile syringe into the loose skin on the lower chest two times a week, alternating sides, for a total of seven weeks (Figure 1). This dose and frequency were chosen based on a previous study of micro-doping performed in humans [12]. This previous study used micro-dosing protocols that change hematological parameters minimally to avoid detection of doping by the ABP [12]. The route of administration was chosen based on previous studies that investigated markers of rHuEPO doping [28]. Horses in the control group received a comparable volume of 0.9% saline subcutaneously at the same time. All animal procedures were approved by the University of California-Davis Institutional Animal Care and Use Committees (IACUC #20319).

### 2.2. Sample Collection

Twenty mL of blood per horse were drawn into EDTA vacutainers immediately prior to initial treatment, three days after initial treatment, once a week for the remainder of seven weeks, and once a week for three weeks following the final rHuEPO treatment.

Blood was also drawn at days 1, 3, 10, 14, 17, 14, 35, 38, 42, 49, 49, 56, and 63 to submit for a complete blood count (CBC) which measured RBCs, hemoglobin, hematocrit, mean corpuscular volume, mean corpuscular hemoglobin, and mean corpuscular hemoglobin concentration. A mixed-effects analysis was used to determine if there was any significant (*p* < 0.05) difference in these parameters between the horses that received saline and those that received rHuEPO.

### 2.3. PBMC and RNA Isolations

Peripheral blood mononuclear cells were isolated from the EDTA blood using Histopaques 1119 and 1077 (Sigmal Aldrich, St. Louis, MO, USA) following the company’s protocol, with the exception that 5 mL of blood was layered onto the histopaques discontinuous gradient and only the mononuclear layer was collected. RNA was isolated immediately after PBMC isolation using a trizol-chloroform phase separation followed by a column clean up (Zymo, Irvine, CA, USA).

### 2.4. RNA Sequencing and Analysis

RNA samples from all horses from day 0, day 3, week 1, week 4, and week 7 were chosen for sequencing. All RNA had an RNA integrity number >7. Strand-specific libraries (Universal Plus mRNA) were created and sequenced on an Illumina NovaSeq to a targeted depth of 30 million reads/sample.

Raw reads were trimmed with trimmomatic [29] to remove low quality reads and adapter sequences. The read were mapped to EquCab3.0 using STAR aligner [30], and the RefSeq annotation (GCF_002863925.1_EquCab3.0). Count data were normalized with transcript per million mapped read normalization in EdgeR (version 3.34.0) [31], and limma-voom (3.48.1) [32] was used to analyze differential expression. Transcripts that were significantly different between treatment groups across two time points were identified using a model including factors for the treatment group, time point (as a categorical variable), the interaction between treatment and time point, and sex. These transcripts were then prioritized to those that were significantly different across at least two time point comparisons. Reported *p*-values were adjusted via the Benjamini-Hochberg false discovery rate (FDR), with *P_FDR_* < 0.05 considered significant.

In order to compare RNA-seq results to those obtained from the RT-qPCR study, counts per million (CPMs) for the top three differentially expressed transcripts were transformed by taking the log_2_ of the CPM+1 and then graphed. A mixed-effects model, where the horse was a random effect and time point, treatment, and their interaction were fixed, was used to confirm significant differences between treatment groups. GraphPad Prism (GraphPad Software, San Diego, CA, USA, www.graphpad.com, accessed on 28 June 2021) was used to create the model and perform post-hoc multiple comparison testing. Stringtie [33] was used to determine if there were multiple isoforms of the three transcripts of interest present in the RNA-seq.

### 2.5. RT-q PCR Validation

To validate the top transcripts across all time points, primers were designed to span two exons of the target transcripts (Supplementary Table S1). The RNA-seq data was used to design the primers and ensure reads were present at the primer locations. The same aliquot of RNA (1200 ng) that was used for RNA-seq from each horse at every time point was reverse transcribed into cDNA with Superscript III (Thermo Fisher Scientific, Waltham, MA, USA). Reverse transcriptase quantitative PCR (RT-qPCR) was performed in triplicate using SYBR green on an AriaMx Real-Time PCR System (Agilent, Santa Clara, CA, USA). The housekeeping gene *B2M* was used for normalization since this reference gene has been shown to have stable expression in PBMCs [34,35]. *ACTB* was also used as a reference gene to confirm results found with *B2M.* The ΔΔCt values and fold changes were calculated to determine differential transcript expression between treatment groups and time points. A 2-way repeated measures ANOVA was used to identify if there were any significant differences between saline and rHuEPO treated groups or time points.

## 3. Results

### 3.1. Horses

All horses successfully completed both the exercise and micro-dosing protocol, except for one horse (#790) in the treatment group that colicked after rHuEPO administration ended and temporarily stopped the exercise protocol. There is no evidence that the colic was related to rHuEPO administration. Administration of subcutaneous rHuEPO did result in apparent discomfort in 5/6 horses after each injection, as indicated by pawing after the injection, leg swelling in one horse, and hives and swelling or edema at the injection site in four horses the day after injection. There was no evidence of abscessation at the injection site and the discomfort only occurred in the horses that received rHuEPO, suggesting that the product itself caused discomfort.

In comparing the CBC results between treatment groups, no significant differences were observed in any of the parameters measured (Supplementary Figure S1).

### 3.2. RNA Sequencing and Differential Transcript Analysis

Our targeted depth of 30 million reads/sample was achieved, with an average of 37.2 M raw reads and 36.8 M trimmed reads. Multidimensional scale analysis indicated that there was no obvious grouping of samples by time point, treatment, or individual (Supplementary Figure S2).

**Figure 2:**
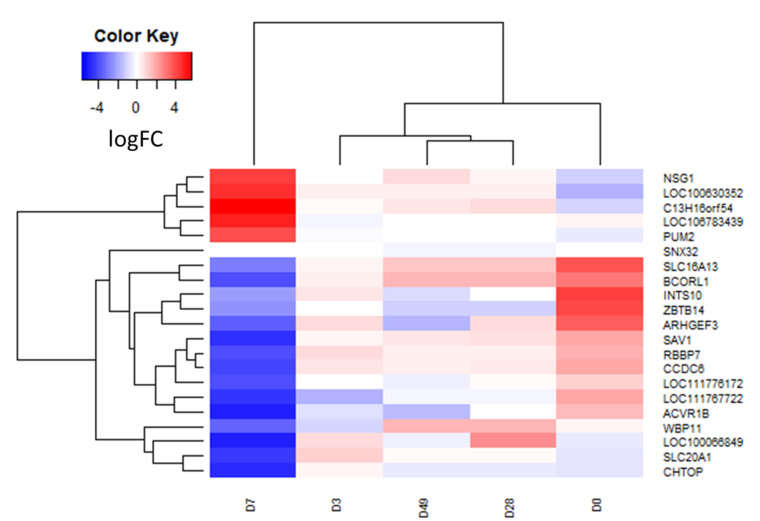
Heat map of the 21 differentially expressed transcripts at each time point. Color is indicative of the log_2_FC, with blue indicating downregulation and red indicating upregulation. Days (D) are organized according to hierarchical cluster on the *x*-axis.

Twenty-one transcripts were determined to be differentially expressed at *P_FDR_* < 0.05 (Table 1, Figure 2). Of the transcripts that were only significant at one time point comparison, 13 were downregulated with rHuEPO administration and 4 were upregulated. Four transcripts (*C13H16orf54* upregulated, *LOC106783439* upregulated than downregulated, *PUM2* upregulated, *CHTOP* downregulated) were significantly dysregulated across two or more time comparisons (Table 1, bolded) and prioritized for RT-qPCR.

### 3.3. RT-qPCR

**Figure 3:**
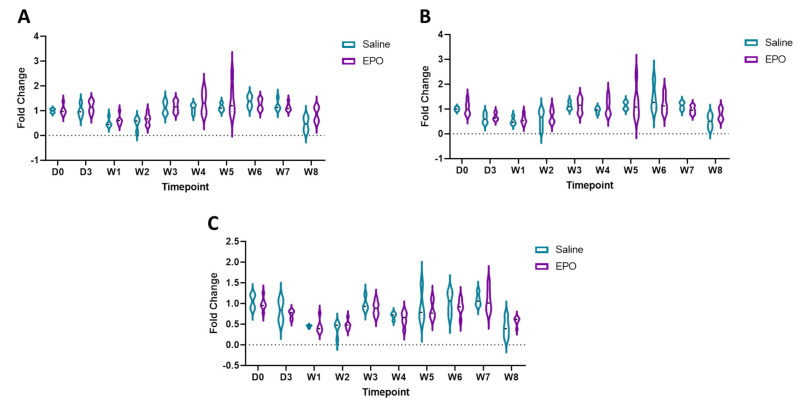
Fold change of (**A**) *C13H16orf54*, (**B**) *PUM2*, and (**C**) *CHTOP* mRNA as determined by RT-qPCR at each time point. Expression is in relation to *B2M*. D stands for day and W indicates week. Each data point is an individual horse, saline treatment as teal and EPO-dosed horses as purple. There was no significant difference of expression between treatment groups.

Of the four prioritized transcripts (Table 1, bolded), one transcript (*LOC106783439*) did not have any exons that aligned between individual horses in our dataset or with the reference exons, indicating an error with the reference. This transcript was thus excluded from further investigation. Primers were successfully designed for the remaining three transcripts (*C13H16orf54*, *PUM2* and *CHTOP*; Table S1) and reference transcript (*B2M*; Table S1). There were no significant differences identified between treatment groups for any of the transcripts of interest via RT-qPCR (Figure 3). *ACTB* was used as a reference gene to confirm these non-significant data, yielding similar results.

To confirm that the results of our model were present in the raw data, we graphed the CPMs (Supplementary Figure S3) of the three transcripts of interest at each time point. After transforming the CPMs, *PUM2* was not significant between treatments, but *C13H16orf54* and *CHTOP* were significantly different at seven days (*p* = 2.9 × 10^−5^, *p* = 0.016, respectively). It was noted that there were several instances where the CPM was zero for each transcript of interest. All libraries passed quality control, so it is unlikely that there is a systemic issue with any of the libraries. However, to be thorough, we also repeated the analysis after removing the zero CPM values (Supplementary Figure S4). As might be expected with decreasing the n, *CHTOP* was no longer significant at seven days (*p =* 0.26). Since all the *C13H16orf54* saline CPMs are zero at day seven and were removed, we do not see any significant difference between the treatment groups at any time point.

We hypothesized that there may be multiple isoforms of the transcripts of interest and that differential expression of the transcripts is what is leading to the conflicting results between the RNA-seq and RT-qPCR. We determined that, in our data set, there were nine *PUM2* isoforms and five isoforms of *CHTOP*. Seventeen of the possible 22 exons in *PUM2* are conserved across all transcripts present in the data set and, in *CHTOP*, there are 4 exons conserved across all transcripts, with seven total possible exons. The primers for RT-qPCR were designed to amplify exons that were conserved across the majority of isoforms. Primers to amplify *PUM2* spanned exons 6–7, and primers for *CHTOP* spanned exons 2–3. However, this prevented the detection of different isoforms.

## 4. Discussion

This study set out to identify transcriptomic markers of rHuEPO micro-doping in Thoroughbred racehorses. Like endogenous EPO, rHuEPO binds to the EPO receptor on erythroid progenitor cells, initiating a signaling cascade that leads to the binding of key transcription factors that induce the production of more red blood cells [36]. Increasing the number of red blood cells increases the total oxygen available and aerobic power [1]. Thus, we hypothesized that we would identify significantly differential transcripts that were involved in erythropoiesis.

RNA-seq from PBMCs isolated throughout the experiment identified three transcripts that changed significantly over time between treatment groups. *C13H16orf54* and *PUM2* were upregulated and *CHTOP* was downregulated with rHuEPO administration. The function of *C13H16orf54* is unknown, but in humans, it is primarily expressed in whole blood [37]. Slightly more is known about the involvement of *PUM2* and *CHTOP* in erythropoiesis. *PUM2*, an RNA-binding protein, has been shown to have a role in hematopoietic stem cell survival and proliferation [38]. Hematopoietic stem cells are progenitor cells to erythroid stem cells that develop into red blood cells [39], so it follows that *PUM2* changes with rHuEPO dosing. *CHTOP* is an important regulator of γ-globin gene expression, which is a fetal globin gene [40]. A previous small study administered rHuEPO to patients with sickle cell anemia and β-thalassemia and identified an increase in their γ-globin levels [41]. rHuEPO has been further investigated in the treatment of β-thalassemia patients in combination with the fetal globin gene inducer butyrate [42]. A pilot study demonstrated that the rHuEPO was required in some patients to respond to butyrate [42], indicating that rHuEPO and γ-globin, can work synergistically to increase total hemoglobin concentrations. Our study further supports the relationship between rHuEPO dosing and γ-globin expression.

With the significant gene expression differences not validated using RT-qPCR, the development of a diagnostic test to detect rHuEPO doping in racehorses is hindered. Although the same RNA was used for both RNA-seq and RT-qPCR, RT-qPCR is subject to more variability [43]. Complementary cDNA synthesis and use of a reference gene in particular add to the variability of the technology [43]. Additionally, since both *PUM2* and *CHTOP* have several known transcript isoforms, specific isoforms may be differentially expressed with rHuEPO administration. In our data set, there were nine *PUM2* isoforms and five isoforms of *CHTOP*. The RT-qPCR primers were designed to target the exons that were most conserved throughout the isoforms. However, this might have masked significant isoforms that were detected by the more sensitive RNA-seq. This poses a point of consideration for future studies evaluating transcriptomic markers of doping.

It is noteworthy that the CPMs are zero for the transcripts of interest in several samples at one or more time points. Since the subsequent RT-qPCR indicated that there is actually transcript present, this brings into question whether there was a technical error. It is unlikely an issue with the RNA itself since the same RNA was used for both RNA-seq and RT-qPCR. To address this concern, the analysis was repeated with only the non-zero CPMs. While there was no longer any significant difference between treatment groups at day 7, this is most likely due to a decrease of n. Day seven had to be completely removed from analysis for *C13H16orf54* since all the CPMs from the saline group were zero, and over half of the individuals were removed in the analysis of *CHTOP*. However, the trend is still present in *CHTOP*. If a test using these transcripts to detect doping is pursued further, these zero CPMs should be further investigated to determine if the cause is biological or technical.

PBMCs were chosen as the cells of interest to identify transcriptomic changes since they are present in the blood samples that are already routinely collected from racehorses. PBMCs have EPO receptors that can be modulated by EPO treatment in humans [27]. However, since the PBMC transcriptomic changes identified do not translate to an easy commercial test, reticulocytes may be better for future studies on rHuEPO micro-dosing since reticulocytes increase with rHuEPO treatment [44], and thus the transcriptomic changes may be more robust.

A limitation to this study is the small sample size of ten horses, with six administered rHuEPO and four salines. Six horses have been shown to be a sufficient population size to identify significantly differential gene expression in horses [45]. In an effort to identify differentially expressed transcripts with a limit of ten horses, the horses were separated into unbalanced groups. However, our results require validation in another larger population of exercising Thoroughbreds. Furthermore, the dosing regimen selected for this study was designed based on previous studies performed on horses and humans [12,28], but it was not evaluated to determine its effect on performance. Red blood cell count, hemoglobin, and hematocrit were measured to determine if the parameters were affected by the micro-doses. While there was a trend of increased values in the middle of the study, there was no overall significant effect of treatment on the parameters. However, this is not unexpected since the dosing regimen was derived from a study that aimed to use a protocol that evaded detection by the ABP [12].

Another factor to consider in the development of a test for transcriptomic markers of doping is that the three transcripts identified were significantly different between treatment groups at only a subset of the time point comparisons. This limits the ability for these transcripts to be reliable markers of rHuEPO micro-dosing. Markers that can be detected at range of time after administration will be the most useful, so this should be taken into account in future experiments looking at transcriptomic markers of doping. It may necessitate the use of more horses to identify more reliable transcriptomic markers that are able to detect rHuEPO at a wide range of time points.

A previous study investigated transcriptomic markers in whole blood of a full dose regimen of rHuEPO in horses [28]. Due to differences in the bioinformatics analysis and updates to the equine reference genome, only two transcripts found to be significant in this past study were present in our dataset (*RPL14*, *BSG*). Neither of these transcripts were significant at any time point comparison between the two treatment groups. Another group also identified 15 transcriptomic markers of micro-doses of rHuEPO in whole blood from human athletes using an adaptive model. Of these, eight were present in our data set (*BCL2L1*, *DCAF12*, *EPB42*, *SELENBP1*, *TMOD1*, *FAM46C*, *STRADB*, and *UBXN6*), but none were significantly differentially expressed between the treatment groups at any time point comparison. A likely reason for the differences in results between our study and previous studies is that we used RNA from PBMCs instead of whole blood to decrease the number of total transcripts and thus increase our chances of identifying significant changes between treatment groups.

With the many different methods that have been found to directly detect exogenous EPO [21,22,23,24,25,26], the importance of identifying transcriptomic markers of rHuEPO doping may not be immediately apparent. However, the downstream effects of rHuEPO treatment likely are similar to the effects of other erythropoiesis-stimulating agents (ESAs). Identifying and testing for these transcriptomic changes may make it easier to identify new drugs as they enter the racing industry. Our study demonstrated the largest number of differentially expressed transcripts when comparing day 0 and day 7. Thus, transcriptomic changes may be detectable longer than the 2–3 days that is typical for the current technologies [21,22,23,24,25].

To develop a test to identify transcriptomic markers of rHuEPO dosing, ddPCR and micro-RNA should be investigated. Droplet digital PCR has been shown to be more sensitive than RT-qPCR [46], and thus could potentially detect the changes identified by RNA-seq. Additionally, ddPCR has been successfully used to detect differential expression of different isoforms of the same transcript in the horse [47]. *PUM2* and *CHTOP* have several known isoforms, warranting additional investigation into expression differences between isoforms with rHuEPO micro-doses. Additionally micro-RNA have started to be investigated in relation to doping in horses [48]. These are promising biomarkers as they are often more resistant to degradation than mRNA [49], and should be further studied as indicators of doping.

Recent, highly-publicized positive drug results in horse racing have highlighted the need for reliable tests. While these were not identified as rHuEPO, preventing any illicit drug doping protects the health and safety of both horses and jockeys, and it protects the integrity of a sport that has a substantial betting culture. A case study was published reporting on two Thoroughbreds that developed anti-rHuEPO antibodies which ended up cross-reacting with endogenous EPO, decreasing erythropoiesis and causing anemia [50]. While the risk of this happening more broadly is not known, it is further motivation to deter the use of rHuEPO as a performance enhancing drug.

In summary, we identified two transcripts that were significantly upregulated and one transcript that was significantly downregulated by RNA-seq analysis in horses administered micro-doses of rHuEPO. The changes in *C13H16orf54*, *PUM2* and *CHTOP* provide insight into the effects of rHuEPO dosing. Since these differences were not detectable via RT-qPCR, these transcripts are not suitable biomarkers of rHuEPO micro-doping. Further work is required to validate these findings and determine the optimal way to use transcriptomic data to inform micro-doping detection tests.

## Figures and Tables

**Table 1:** Transcripts significantly differentially expressed between treatment groups (EPO vs. control) across two time points. D = day, log_2_FC = log_2_ of fold change, AveExpr = average expression across all samples, *p*.Value = raw *p*-value, adj.*p*.Val = FDR corrected *p*-value. Bolded transcripts are those that are significantly differentially expressed at more than one time comparison.

Time Point	Gene Name	Log_2_FC	Ave Expr	*p* Value	adj. *p*. Val
D7 v D0	BCORL1	−6.91	3.58	1.04 × 10^−6^	7.90 × 10^−3^
	**C13H16orf54**	6.68	3.98	1.48 × 10^−6^	7.90 × 10^−3^
	LOC100630352	6.32	5.13	2.25 × 10^−6^	8.04 × 10^−3^
	SLC16A13	−6.61	4.45	7.80 × 10^−6^	1.53 × 10^-2^
	ARHGEF3	−7.18	3.39	1.06 × 10^−5^	1.53 × 10^−2^
	LOC111776172	−4.96	5.93	1.11 × 10^−5^	1.53 × 10^−2^
	**LOC106783439**	4.70	5.05	1.14 × 10^−5^	1.53 × 10^−2^
	LOC111767722	−6.38	2.94	1.15 × 10^−5^	1.53 × 10^-2^
	**PUM2**	4.40	5.19	1.28 × 10^−5^	1.53 × 10^−2^
	CCDC6	−6.17	2.92	1.43 × 10^−5^	1.53 × 10^−2^
	INTS10	−6.56	2.85	1.61 × 10^−5^	1.57 × 10^−2^
	SAV1	−6.48	3.80	2.74 × 10^−5^	2.45 × 10^−2^
	RBBP7	−5.70	4.16	3.41 × 10^−5^	2.80 × 10^−2^
	ACVR1B	−6.41	3.24	3.66 × 10^−5^	2.80 × 10^-2^
	**CHTOP**	−4.03	4.68	4.42 × 10^−5^	3.15 × 10^−2^
	NSG1	5.34	3.92	7.67 × 10^−5^	4.98 × 10^−2^
	ZBTB14	−6.47	1.41	7.91 × 10^−5^	4.98 × 10^−2^
D7 v D3	**LOC106783439**	5.31	5.05	2.76 × 10^−6^	2.42 × 10^−3^
	**CHTOP**	−5.00	4.68	4.53 × 10^−7^	2.42 × 10^−3^
	**PUM2**	4.11	5.19	1.21 × 10^−5^	4.32 × 10^-2^
	**C13H16orf54**	5.55	3.98	2.08 × 10^−5^	4.32 × 10^−2^
	SLC20A1	−5.43	3.42	2.17 × 10^−5^	4.32 × 10^−2^
D28 v D0	SNX32	−0.39	13.85	9.48 × 10^−7^	1.01 × 10^−2^
D28 v D7	**LOC106783439**	−5.08	5.05	2.90 × 10^−7^	3.10 × 10^−3^
	LOC100066849	7.49	2.30	1.32 × 10^−6^	7.08 × 10^−3^
	WBP11	5.08	5.08	3.27 × 10^−6^	1.07 × 10^−2^
	**CHTOP**	4.24	4.68	1.25 × 10^−5^	3.35 × 10^−2^
D49 v D7	**LOC106783439**	−5.90	5.05	7.55 × 10^−9^	8.08 × 10^−5^

## Data Availability

The mapped bam files are available on NCBI’s sequence read archive (PRJNA769732).

## Supplementary Materials

The following are available online at https://www.mdpi.com/article/10.3390/genes12121874/s1, Table S1: Primer sequences, Figure S1: Complete blood count results, Figure S2: Multidimensional scaling (MDS) plots of read counts normalized by EdgeR, Figure S3: CPMs transformed by log_2_(CPM+1), Figure S4: Non-zero CPMs transformed by log_2_(CPM+1).
