# Supplementary material for: Transcriptomic Markers of Recombinant Human Erythropoietin Micro-Dosing in Thoroughbred Horses
Source: Genes (Basel). 2021 Nov 24;12(12):1874. doi: 10.3390/genes12121874 (PMC8702184; doi:10.3390/genes12121874)
Supplement: Supplementary file 1 [file genes-12-01874-s001.zip › genes-1447398-supplementary.pptx]

## Slide 1
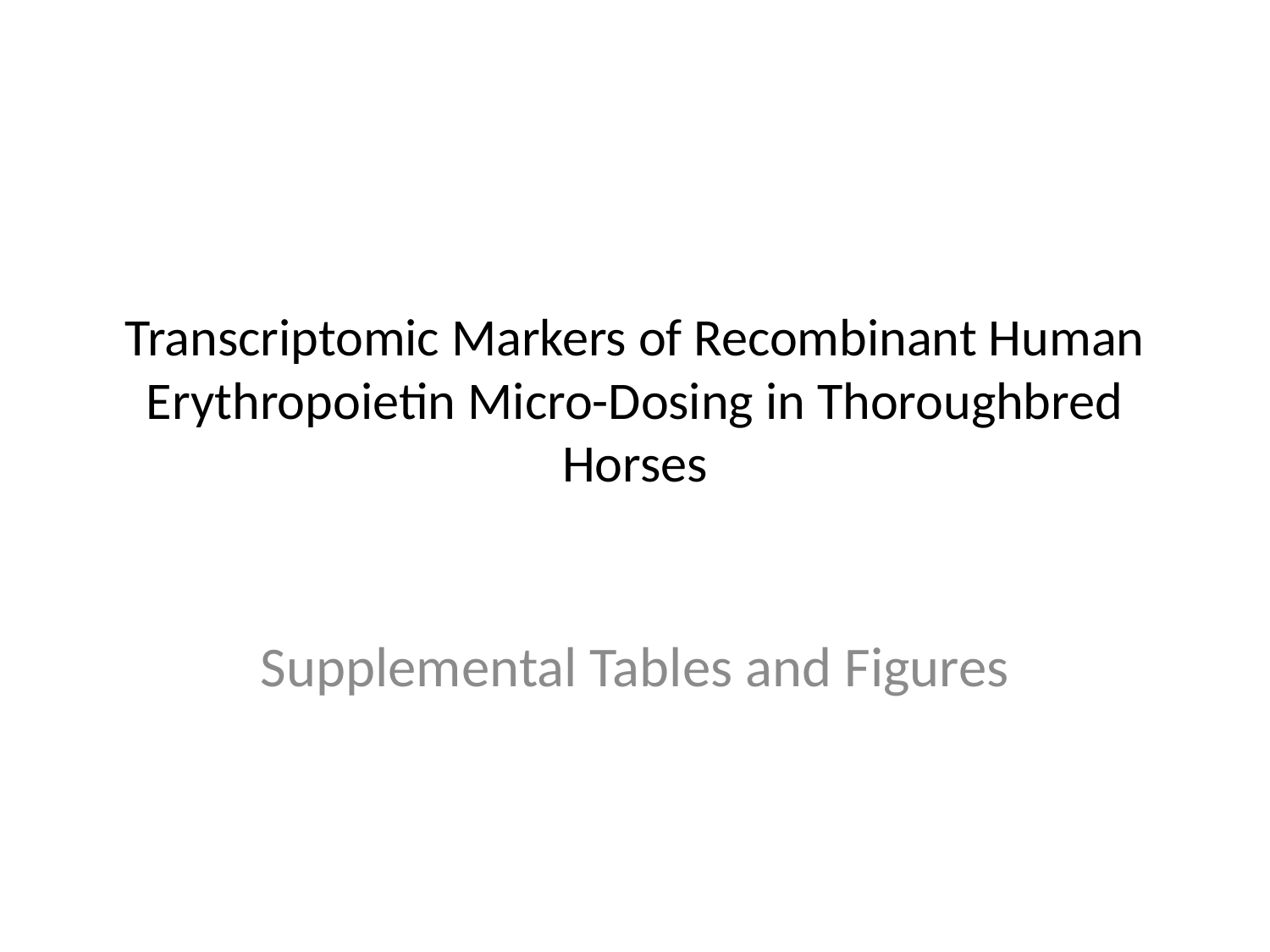

# Transcriptomic Markers of Recombinant Human Erythropoietin Micro-Dosing in Thoroughbred Horses
Supplemental Tables and Figures

## Slide 2
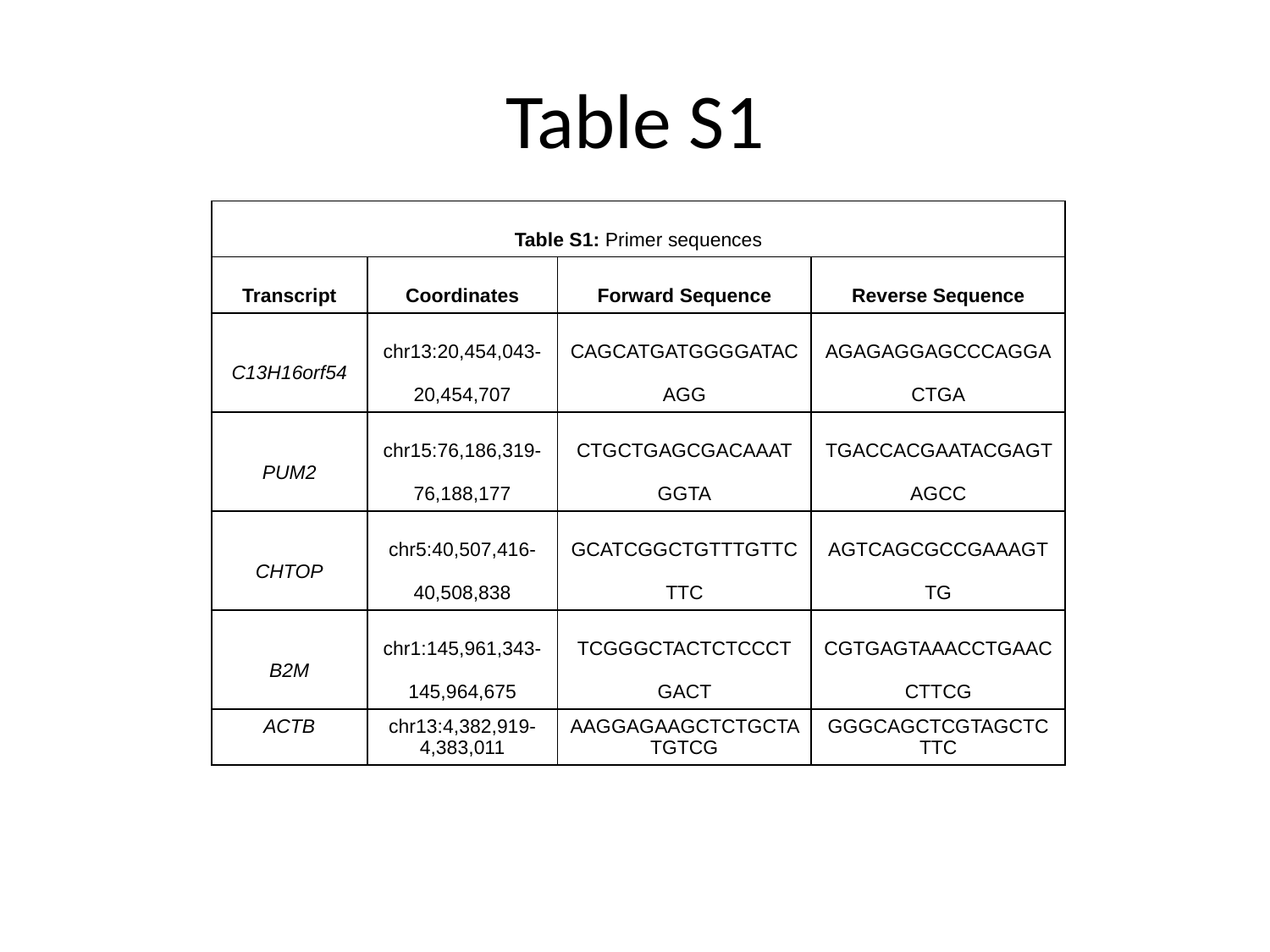

# Table S1
| Table S1: Primer sequences | | | |
| --- | --- | --- | --- |
| Transcript | Coordinates | Forward Sequence | Reverse Sequence |
| C13H16orf54 | chr13:20,454,043-20,454,707 | CAGCATGATGGGGATACAGG | AGAGAGGAGCCCAGGACTGA |
| PUM2 | chr15:76,186,319-76,188,177 | CTGCTGAGCGACAAATGGTA | TGACCACGAATACGAGTAGCC |
| CHTOP | chr5:40,507,416-40,508,838 | GCATCGGCTGTTTGTTCTTC | AGTCAGCGCCGAAAGTTG |
| B2M | chr1:145,961,343-145,964,675 | TCGGGCTACTCTCCCTGACT | CGTGAGTAAACCTGAACCTTCG |
| ACTB | chr13:4,382,919-4,383,011 | AAGGAGAAGCTCTGCTATGTCG | GGGCAGCTCGTAGCTCTTC |

## Slide 3
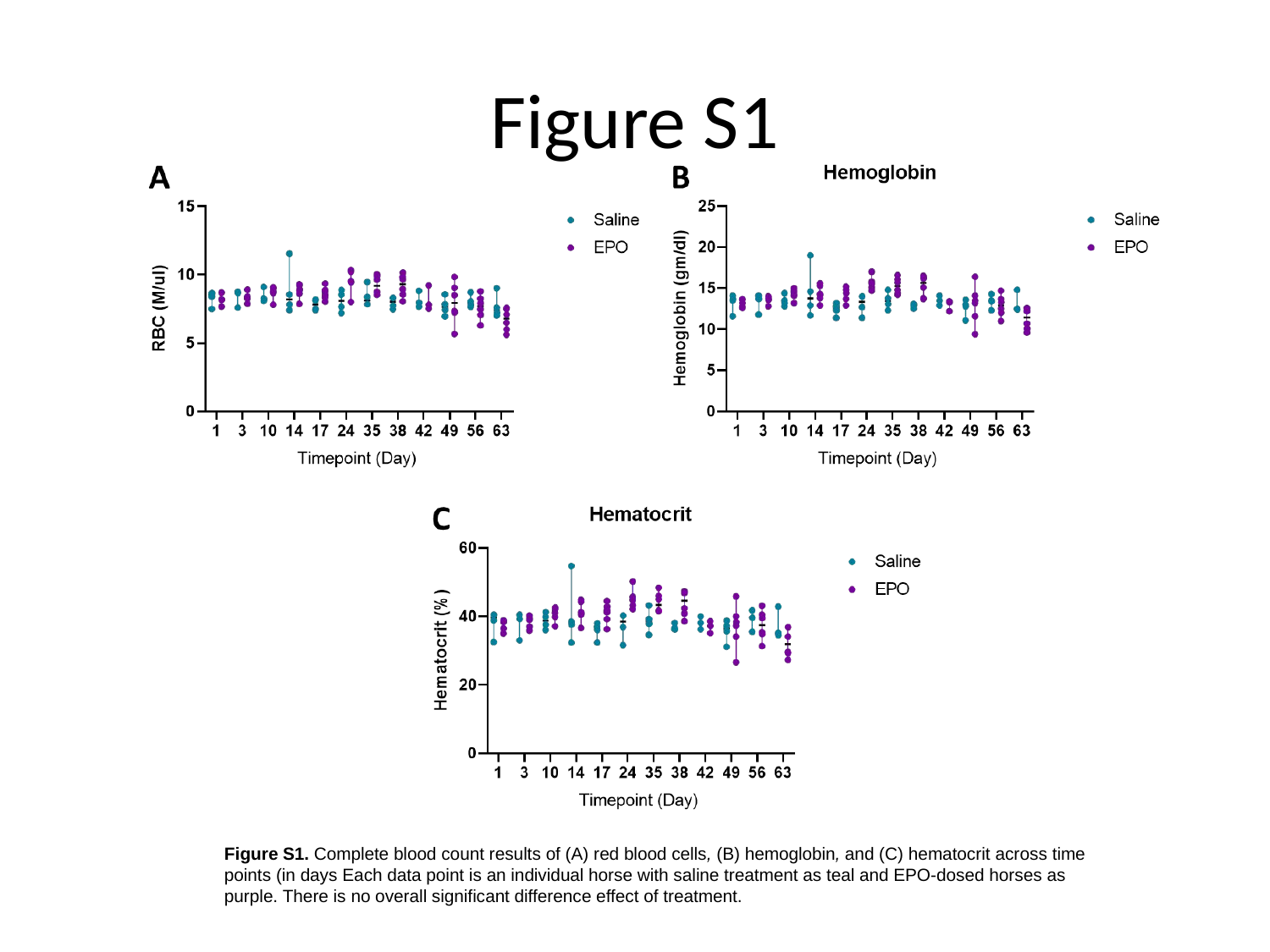

# Figure S1
Figure S1. Complete blood count results of (A) red blood cells, (B) hemoglobin, and (C) hematocrit across time points (in days Each data point is an individual horse with saline treatment as teal and EPO-dosed horses as purple. There is no overall significant difference effect of treatment.

## Slide 4
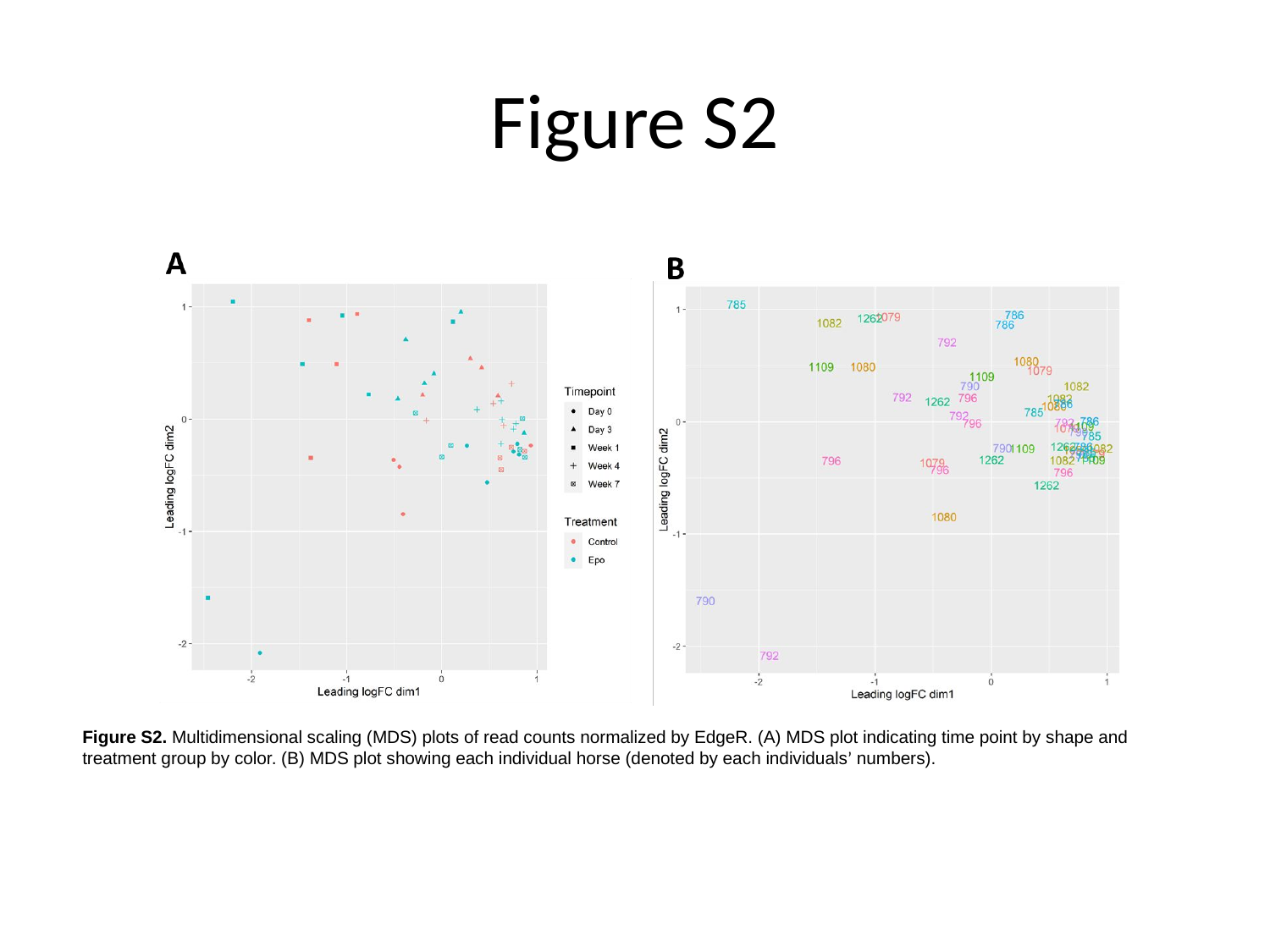

# Figure S2
Figure S2. Multidimensional scaling (MDS) plots of read counts normalized by EdgeR. (A) MDS plot indicating time point by shape and treatment group by color. (B) MDS plot showing each individual horse (denoted by each individuals’ numbers).

## Slide 5
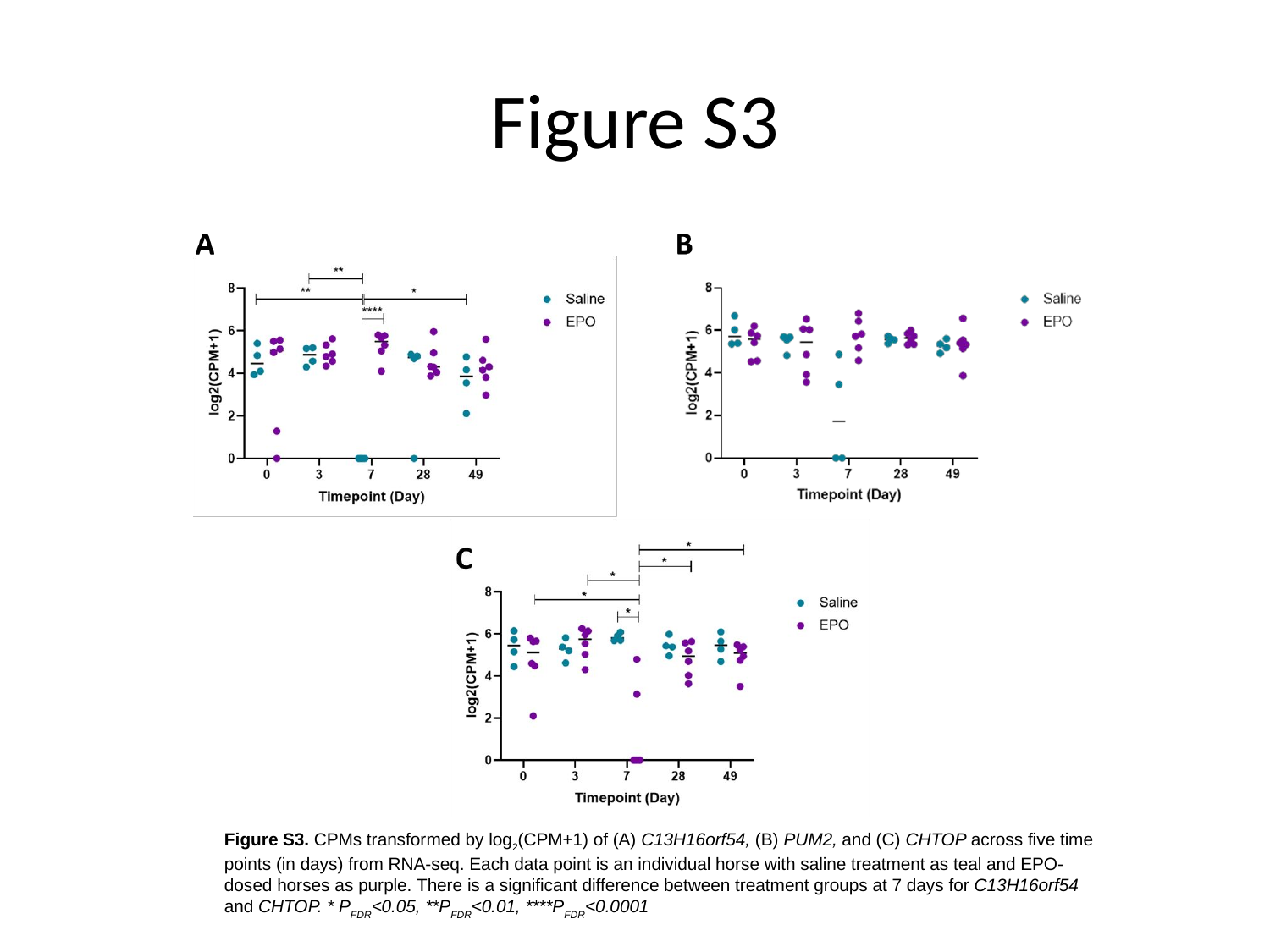

# Figure S3
Figure S3. CPMs transformed by log2(CPM+1) of (A) C13H16orf54, (B) PUM2, and (C) CHTOP across five time points (in days) from RNA-seq. Each data point is an individual horse with saline treatment as teal and EPO-dosed horses as purple. There is a significant difference between treatment groups at 7 days for C13H16orf54 and CHTOP. * PFDR<0.05, **PFDR<0.01, ****PFDR<0.0001

## Slide 6
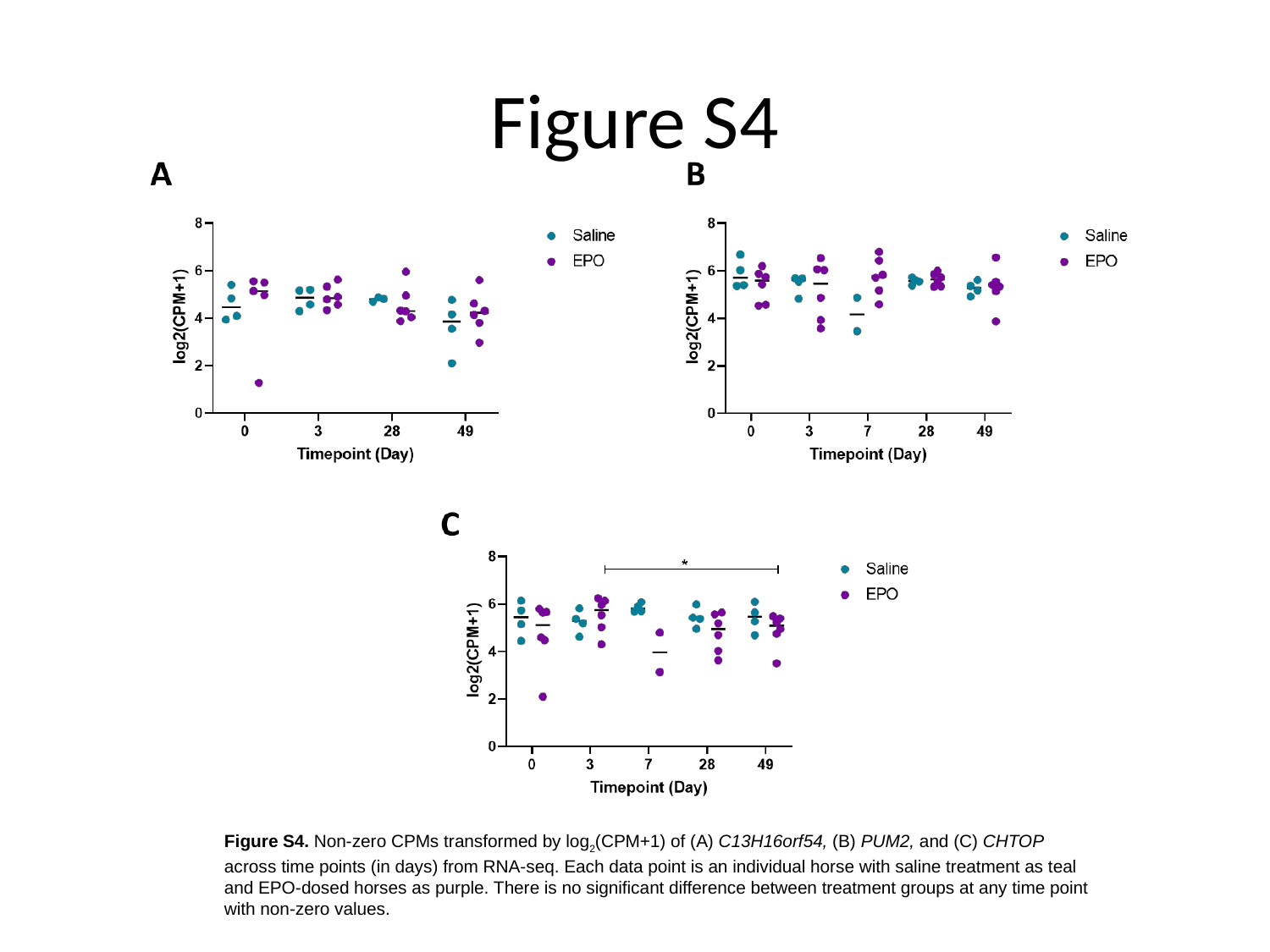

# Figure S4
Figure S4. Non-zero CPMs transformed by log2(CPM+1) of (A) C13H16orf54, (B) PUM2, and (C) CHTOP across time points (in days) from RNA-seq. Each data point is an individual horse with saline treatment as teal and EPO-dosed horses as purple. There is no significant difference between treatment groups at any time point with non-zero values.
